# Multiplex ligation-dependent probe amplification versus karyotyping in prenatal diagnosis: the M.A.K.E. study

**DOI:** 10.1186/1471-2393-8-18

**Published:** 2008-05-20

**Authors:** Elisabeth MA Boormans, Erwin Birnie, Hajo I Wildschut, Heleen G Schuring-Blom, Dick Oepkes, Carla AC van Oppen, Jan G Nijhuis, Merryn VE Macville, Angelique JA Kooper, Karin Huijsdens, Mariëtte VJ Hoffer, Attie Go, Johan Creemers, Shama L Bhola, Katia M Bilardo, Ron Suijkerbuijk, Katelijne Bouman, Robert-Jan H Galjaard, Gouke J Bonsel, Jan MM van Lith

**Affiliations:** 1 Department of Obstetrics and Gynecology, Academic Medical Centre, Amsterdam, The Netherlands; 2 Department of Obstetrics and Gynecology, Onze Lieve Vrouwe Gasthuis, Amsterdam, The Netherlands; 3 Institute of Health Policy and Management, Erasmus Medical Center, Rotterdam, The Netherlands; 4 Department of Obstetrics and Gynecology, Erasmus Medical Center, Rotterdam, The Netherlands; 5 Department of Medical Genetics, University Medical Center Utrecht, Utrecht, The Netherlands; 6 Department of Obstetrics and Gynecology, Leiden University Medical Center, Leiden, The Netherlands; 7 Department of Obstetrics and Gynecology, University Medical Center Utrecht, Utrecht, The Netherlands; 8 Department of Obstetrics and Gynecology, Academic Medical Center Maastricht, Maastricht, The Netherlands; 9 Department of Clinical Genetics, Academic Medical Center Maastricht, Maastricht, The Netherlands; 10 Department of Human Genetics, Radboud University Nijmegen Medical Center, Nijmegen, The Netherlands; 11 Department of Clinical Genetics, Academic Medical Center, Amsterdam, The Netherlands; 12 Department of Clinical Genetics, Leiden University Medical Center, Leiden, The Netherlands; 13 Department of Obstetrics and Gynecology, VU Medical Center, Amsterdam, The Netherlands; 14 Department of Obstetrics and Gynecology, Radboud University Nijmegen Medical Center, Nijmegen, The Netherlands; 15 Department of Clinical Genetics, VU Medical Center, Amsterdam, The Netherlands; 16 Department of Clinical Genetics, University Medical Center Groningen, Groningen, The Netherlands; 17 Department of Clinical Genetics, Erasmus Medical Center, Rotterdam, The Netherlands

## Abstract

**Background:**

In the past 30 years karyotyping was the gold standard for prenatal diagnosis of chromosomal aberrations in the fetus. Traditional karyotyping (TKT) has a high accuracy and reliability. However, it is labor intensive, the results take 14–21 days, the costs are high and unwanted findings such as abnormalities with unknown clinical relevance are not uncommon. These disadvantages challenged the practice of karyotyping. Multiplex ligation-dependent probe amplification (MLPA) is a new molecular genetic technique in prenatal diagnosis. Previous preclinical evidence suggests equivalence of MLPA and traditional karyotyping (TKT) regarding test performance.

**Methods/Design:**

The proposed study is a multicentre diagnostic substitute study among pregnant women, who choose to have amniocentesis for the indication advanced maternal age and/or increased risk following prenatal screening test. In all subjects, both MLPA and karyotyping will be performed on the amniotic fluid sample. The primary outcome is diagnostic accuracy. Secondary outcomes will be maternal quality of life, women's preferences and costs. Analysis will be intention to treat and per protocol analysis. Quality of life analysis will be carried out within the study population. The study aims to include 4500 women.

**Discussion:**

The study results are expected to help decide whether MLPA can replace traditional karyotyping for 'low-risk' pregnancies in terms of diagnostic accuracy, quality of life and women's preferences. This will be the first clinical study to report on all relevant aspects of the potential replacement.

**Trial Registration:**

The protocol is registered in the clinical trial register number ISRCTN47252164

## Background

In the Netherlands, invasive prenatal diagnosis is offered to all women who are considered to be at increased risk for Down's syndrome. Until recently, the vast majority of invasive tests was done for advanced maternal age. More recently, a nationwide screening program for Down's syndrome using first trimester serum testing combined with ultrasonographic nuchal translucency measurement was introduced [1,2]. A positive result of this combination test or maternal age (36 years or older) represent 80% of the indications for the invasive diagnostic procedures (amniocentesis and chorionic villus sampling). We have seen a still continuing shift of maternal age-based karyotyping towards prenatal screening based testing.

Traditional karyotyping (TKT) is considered the gold standard for invasive prenatal diagnosis (PND) [2,3]. TKT is able to detect a range of numerical and structural chromosomal abnormalities with considerable accuracy (99.4–99.8%) and reliability[3,4]. However, TKT also has several disadvantages: it is labour intensive and the costs are high. Furthermore, obtaining results from karyotyping takes 2–3 weeks, and this waiting time places a significant emotional burden on women and their partners[5]. Moreover, the extensive detection capacity of TKT can be perceived as a disadvantage due to the detection of abnormalities with unclear or mild clinical relevance. These results can cause patient anxiety, emotional dilemmas concerning the continuation of pregnancy and, albeit rare, unnecessary pregnancy terminations [6].

Due to these disadvantages, TKT has been challenged as a reference test. In 2002, a molecular PCR-based technique, MLPA (multiplex ligation-dependent probe amplification) became available to detect foetal aneuploidies in amniotic fluid cells. In preclinical laboratory studies, MLPA has been a robust test in detecting the most common chromosomal aneuploidies, namely trisomy 21, 13, 18 and sex chromosome abnormalities [7-9]. Compared to TKT, MLPA has several potential advantages: the result is available in 2–4 days instead of 3 weeks, the technique requires only 2 ml of amniotic fluid instead of 16–20 ml, and the technique is considerably less labour-intensive and is suitable for high-throughput testing [8-10].

In the Netherlands, like other western countries, there is an ongoing debate whether rapid molecular tests should be used as a stand-alone diagnostic tool instead of karyotyping in prenatal diagnosis for certain indications[5,11-15]. To strengthen the debate and to supply it with evidence, we designed a clinical prospective cohort study in which MLPA is compared independently to TKT for the two main referral indications; advanced maternal age and increased risk following prenatal screening tests. The aim is to estimate diagnostic accuracy for the detection of trisomies 21, 18,13 and sex chromosome abnormalities, as well as the reduction in waiting time for the prospective parents, maternal quality of life, women's preferences, the relevance of 'unexpected findings' and costs.

## Methods/Design

### Aims

The M.A.K.E. study investigates whether MLPA can replace TKT in prenatal diagnosis in terms of diagnostic accuracy, costs and maternal quality of life for women undergoing amniocentesis for the indications advanced maternal age and increased risk following prenatal screening.

We hypothesize that MLPA has equivalent diagnostic accuracy in detecting trisomies 21, 18, 13 and sex chromosome abnormalities. The reduction in waiting time for the patient, maternal quality of life, women's preferences, the relevance of 'unexpected findings' and costs are part of the study.

### Study design

The M.A.K.E. study is a prospective, nationwide multicenter study. All eight genetic centres in the Netherlands will participate.

### Participants

Participants are pregnant women choosing to have amniocentesis for either advanced maternal age or increased risk following prenatal screening.

Women undergoing amniocentesis for ultrasound abnormalities, parental chromosomal abnormality, or a previous child with chromosomal abnormality are excluded.

### Procedures, recruitment and collection of baseline data

Pregnant women who visit one of the participating antenatal care units are informed by an obstetrician, midwife or genetic counsellor on the different test strategies (prenatal screening and prenatal diagnosis) with associated risks and benefits of each procedure. After they have chosen to have amniocentesis, eligible women will receive participation information. After written consent, amniotic fluid is obtained and tested with both MLPA and karyotyping. Stratification will be applied for each centre. Baseline demographic characteristics and obstetric history are recorded. For the quality of life study and women's preference study a representative sub sample is drawn, with separate consent procedure.

### Diagnostic tests

Details on amniocentesis are recorded in the case record form, accessible through a secure website [16]. All amniotic fluid samples are tested with both MLPA and TKT. Test results are blindly assessed. We chose not to fully standardize the MLPA and TKT protocols but to accept the minor differences within the eight laboratories in sample preparation, formulation of the trisomy multiplex, equipment and interpretation of results. This will make the trial more pragmatic, with better generalizability of the results. The existing differences will be administered and will be helpful in choosing the best test strategy in the nearby future.

#### MLPA

In this study we use a commercially available kit, SALSA MLPA P095 (MRC Holland, the Netherlands). For each specific genomic target, a set of 2 probes is designed to hybridize immediately adjacent to each other on the same target strand. Both probes consist of a short target specific sequence and a universal PCR primer-binding site. One of the probes contains a so called 'stuffer sequence'. For each probe in the multiplex, the stuffer part has a specific length and sequence. After overnight hybridization to the target DNA, each pair of adjacent probes is joined by a ligation reaction. Next, PCR is performed with a single fluorescent-labelled primer pair, which ensures that the relative yield of each of the PCR products is proportional to the amount of each of the target sequences. The different length products are separated on an automated capillary sequencer and the peak areas are quantified[9].

Compared to other techniques available for rapid aneuploidy detection (RAD), i.e. quantitative fluorescence polymerase chain reaction (QF-PCR) and fluorescence in situ hybridization (FISH), MLPA has the advantage of detecting 40 different loci in a single reaction. Furthermore, MLPA is suitable to tailor-made strategies for different clinical settings. Compared to FISH, MLPA is suitable for high-throughput testing and less expensive. Compared to QF-PCR, MLPA avoids the problem of non-informativeness of the polymorphic markers that may occur with QF-PCR [17]. However, in contrast to QF-PCR, MLPA will not diagnose 69, XXX triploidy.

#### Karyotyping

Karyotyping is a reliable and accurate test for detecting chromosomal aberrations. The karyotyping detection rate for Down syndrome is 99,5% [3].

The time from amniocentesis to diagnosis is 2–3 weeks, as the test is non-automated and samples need to be cultured in order to obtain foetal cells at the metaphase stage. Laboratory personnel involved in TKT are blinded for MLPA results. In case of an abnormal MLPA result and a more rapid test result is wanted, the earliest possible harvesting of cell cultures is carried out.

### Outcome measures

Primary outcome is diagnostic accuracy. Sensitivity, specificity and failure rates of MLPA are calculated for (an)euploidies 21, 13, 18, X and Y and compared with TKT as a reference test. Turnaround time on laboratory and patient level is recorded. Discordant test results and technical difficulties are counted, allowing for general and per centre analysis. The effect of a learning curve if present will be established.

#### Secondary outcomes

Quality of life questionnaires are filled out before amniocentesis and 2, 5, 14, 23 and 63 days after amniocentesis. The questionnaires contain Spielberger's State Trait and Anxiety Inventory (STAI), Impact of Event Scale (IES), Personal Perceived Control (PPC) and Medical Outcome Study Short Form (SF-36) and questions on background characteristics. We will compare two groups, one group, receiving the result of TKT and the other group, receiving results of both MLPA and TKT.

A discrete choice experiment will give insight in patient's preferences and investigates the value pregnant women place on different characteristics of prenatal testing (e.g. detection capacity, waiting time, consequences for the child of the detected abnormalities).

The process of care is distinguished into three cost categories; direct medical costs (all costs in the health care sector), direct non-medical costs (costs outside the health care sector that are affected by health status or health care) and indirect costs of the pregnant women and her partner. For each cost category, costs are measured as the volumes of resources used multiplied with appropriate valuations (cost-per-unit estimates, fees, national reference prices).

### Statistical issues

#### Sample size

Adopting the results of karyotyping as the gold standard and assuming that MLPA and karyotyping do not produce different test results, that not more than 1/500 missed cases of Down syndrome are accepted, and that the proportion of discordant test results is 0.002, we need at least 4500 paired test results to test the null-hypothesis of clinical equivalence (one-sided alpha 0.05, power = 1-beta = 0.90).

#### Data analysis

Data will be analyzed according to both the intention to treat and per protocol analysis. To determine non-inferiority in diagnostic accuracy we will calculate absolute and relative sensitivity, specificity and failure numbers of MLPA and compare these to karyotyping (gold standard). Furthermore, the time cytogeneticists or molecular geneticists need to provide an authorized test results and the time needed to inform the patients is recorded. With these data exact waiting periods for both tests can be estimated.

Quality of life outcomes. Repeated measurement analysis will be performed for the between group evaluation.

Women's preferences. Responses on the discrete choice experiment will be analyzed using conditional logistic regression techniques.

Economic evaluation. Since the study is designed as an equivalence study, we hypothesize there is no difference in diagnostic accuracy between MLPA and TKT. The economic analysis is expected to be a cost-effectiveness analysis with maternal health as effectiveness.

#### Ethical considerations

The study protocol has been approved by the Medical Ethical Committee of the Onze Lieve Vrouwe Gasthuis (06032). The protocol is registered in the clinical trial register number ISRCTN47252164

## Discussion

MLPA can potentially replace the traditional karyotype for detecting the most common chromosomal aneuploidies. This study is designed to provide evidence on clinical equivalency of MLPA compared to TKT in prenatal diagnosis. To determine the best test strategy in prenatal clinical practice for women undergoing amniocentesis for either advanced age or increased risk following prenatal screening, we will investigate diagnostic accuracy, speed, maternal quality of life, women's preferences and costs.

## Abbreviations

MLPA: Multiplex Ligation-dependent Probe Amplification, TKT: Traditional karyotyping, PND: Prenatal diagnosis, RAD: Rapid Aneuploidy Detection, QF-PCR: Quantitative Fluorescent Polymerase Chain Reaction, FISH: Fluorescent *In Situ *Hybridization

## Competing interests

The authors declare that they have no competing interests.

## Authors' contributions

EMAB, EB, GJB and JMMvL were involved in conception and design of the study. EMAB, EB and JMMvL drafted the manuscript. All authors mentioned in the manuscript are members of the M.A.K.E. study group. They participated in the design of the study during several meetings and are local investigators at the participating centres. All authors edited the manuscript and read and approved the final manuscript.

## Pre-publication history

The pre-publication history for this paper can be accessed here:


